# *SF3B1* and *EIF1AX* mutations occur in primary leptomeningeal melanocytic neoplasms; yet another similarity to uveal melanomas

**DOI:** 10.1186/s40478-016-0272-0

**Published:** 2016-01-15

**Authors:** Heidi V. N. Küsters-Vandevelde, David Creytens, Adriana C. H. van Engen-van Grunsven, Marcel Jeunink, Veronique Winnepenninckx, Patricia J. T. A. Groenen, Benno Küsters, Pieter Wesseling, Willeke A. M. Blokx, Clemens F. M. Prinsen

**Affiliations:** Department of Pathology, Canisius Wilhelmina Hospital, P.O. Box 9015, 6500 GS Nijmegen, The Netherlands; Department of Pathology, Ghent University Hospital, De Pintelaan 185, 9000 Ghent, Belgium; Department of Pathology, Radboud University Medical Center, P.O. Box 9101, 6500 HB Nijmegen, The Netherlands; Department of Pathology, Maastricht University Medical Center, P.O. Box 5800, 6202 AZ Maastricht, The Netherlands; Department of Pathology, VU University Medical Center, P.O. Box 7057, 1007 MB Amsterdam, The Netherlands

**Keywords:** Leptomeningeal melanocytic neoplasms, Melanocytoma, Melanoma, Central nervous system, Neurocutaneous melanocytosis, *BAP1*, *SF3B1*, *EIF1AX*

## Abstract

**Introduction:**

Like uveal melanomas, primary leptomeningeal melanocytic neoplasms (LMNs) frequently carry *GNAQ* and *GNA11* mutations. However, it is currently unknown whether these LMNs harbor mutations in *BAP1*, *SF3B1* and/or *EIF1AX* like uveal melanomas as well. In this study, we used Sanger sequencing for the detection of mutations in *SF3B1* (hotspots in exon 14 and 15) and *EIF1AX* (exon 1 and 2 and flanking intronic regions) in a series of 24 primary LMNs. Additionally, BAP1 immunohistochemistry was used as a surrogate marker for the detection of inactivating mutations in the *BAP1* gene.

**Results:**

Mutations in either *SF3B1* or *EIF1AX* were identified in 8 out of 24 primary LMNs (33 %). The presence of these mutations was mutually exclusive and occurred in primary LMNs of different malignancy grades (melanocytomas, intermediate-grade melanocytic tumors, melanomas). Complete absence of nuclear BAP1 staining as is typically seen in *BAP1*-mutated tumors was not observed.

**Conclusions:**

Our finding that an *SF3B1* or *EIF1AX* mutation is present in a substantial subset of primary LMNs underscores that these tumors genetically resemble uveal melanoma and are different from cutaneous melanoma at the genetic level. This information may not only aid in the differential diagnosis of primary versus metastatic melanocytic tumor in/around the central nervous system, but also in the identification of more promising therapeutic approaches targeting the molecular pathways involved in the oncogenesis of LMNs. As none of the primary LMNs in our series showed complete loss of nuclear BAP1 protein, it is unlikely that *BAP1* mutations are frequent in these tumors but the role of this gene warrants further investigation.

## Introduction

Primary leptomeningeal melanocytic neoplasms (LMNs) are infrequent tumors, forming a spectrum ranging from benign or low-grade malignant melanocytomas to frankly malignant melanomas [1]. These tumors share molecular features with uveal melanomas (UMs). In contrast to cutaneous melanomas (CMs), both LMNs and UMs frequently carry mutations in the G protein encoding genes *GNAQ* and *GNA11*, whereas mutations in *BRAF* and in the *TERT* promoter are infrequent [2–8]. This situation reflects the heterogeneous molecular background of different groups of melanoma and has important implications for targeted therapy.

In the past years, inactivating mutations in the tumor suppressor gene *BAP1* (BRCA-associated protein 1) were shown to be implicated in UM [9, 10]. The *BAP1* gene is located on chromosome 3p21.1 and encodes a nuclear ubiquitinase involved in epigenetic modulation of chromatin [11]. Somatic *BAP1* mutations are predominantly present in UMs with monosomy 3 (~85 %), the latter being a strong predictor for metastatic disease [9, 10, 12]. In this setting *BAP1* functions as a tumor suppressor gene, with loss of one copy of chromosome 3 and mutation in the other *BAP1* allele representing the two hits causing inactivation of this gene. Indeed, in UMs with disomy 3 (and a good prognosis), mutations in *BAP1* are rare [6, 9, 10]. A small proportion of patients with UM (~2–3 %) harbor a germline mutation in *BAP1* [13]. These patients suffer from the *BAP1* hereditary cancer syndrome and have an increased risk of developing cutaneous melanocytic tumors as well as a spectrum of non-melanocytic neoplasms including mesothelioma, renal cell carcinoma, meningeoma, and adenocarcinoma of the lung [14, 15]. Very recently, it was suggested that primary leptomeningeal melanoma is part of this cancer predisposition syndrome as well [16].

Furthermore, recurrent hotspot mutations in the *SF3B1* gene (mainly at codon 625) and mutations of the *EIF1AX* gene (spread over exon 1 and 2) were recently reported in UMs, especially in tumors with disomy 3 (up to 30 and 50 % of disomy 3 tumors, respectively) [17–19]. These mutations in UMs appeared to be largely mutually exclusively with *BAP1* mutations, while in CMs these mutations were found to be very infrequent (~1 %) [20].

It is currently unknown whether somatic mutations in *BAP1*, *SF3B1* and *EIF1AX* also characterize primary LMNs. Using Sanger sequencing, we searched for mutations in hotspot regions of *SF3B1* (exon 14 and 15) and exon 1 and 2 and flanking intronic regions of *EIF1AX* in a series of 24 primary LMNs. Additionally, we performed immunohistochemistry for the detection of BAP1 protein loss as a surrogate marker for identification of inactivating *BAP1* mutations.

## Materials and methods

### Patients and histopathology

Formalin fixed and paraffin embedded (FFPE) tissue samples of twenty-four primary LMNs were obtained through the Dutch nationwide histopathology and cytopathology data network and archive (PALGA) [21]. Histology was revised by HK; WHO 2007 criteria were used for classification into melanocytoma, intermediate-grade melanocytic tumor and melanoma [1]. Information on mutation status of *GNAQ*, *GNA11*, *BRAF*, *NRAS* and chromosome 3 status of cases #1–7, #10, #13–18, and #22–24 has been published previously [2, 22, 23]. The study was approved by the local ethics committees of the Canisius Wilhelmina Hospital (ref.nr. LTC/TT/013–2015) and Radboud University (CMO, ref.nr/Dossiernummer: 2015–1692).

### DNA extraction

Representative regions of about five 4-μm-thick FFPE sections with an estimated tumor cell percentage of at least 70 % were manually dissected and used for DNA extraction. After heating in ATL buffer, the tissue sections were incubated in proteinase K for one hour, followed by subsequent purification of the DNA according to the manufacturer (QIAamp DNA Mini Kit, QIAGEN GmbH, Germany). After DNA purification, possible melanin contamination was removed by using an inhibitor removal kit (OneStep™ PCR Inhibitor Removal Kit, Zymo Research). The DNA sample concentration was assessed spectrophotometrically (Varian Cary 50 spectrophotometer, Agilent Technologies). The integrity of the isolated DNA was tested by means of a multiplex ladder PCR [24].

### BAP1 immunohistochemistry

Immunohistochemistry was performed on 4-μm-thick FFPE sections using an automated immunostainer (Benchmark XT, Ventana Medical Systems, Tucson, AZ, USA) according to the manufacturer’s instructions. Sections were immunostained with a primary monoclonal antibody against BAP1 (clone C4, 1:100 dilution, Santa Cruz Biotechnology, Dallas, TX, USA) using 3,3-diaminobenzidine (DAB) as chromogen. Selected cases that showed a staining result that was difficult to interpret including strongly pigmented tumors (patients #1–8, #12–16, #22–24) were manually stained (clone C4, 1:50 dilution, Immunologic, Duiven, the Netherlands) according to the manufacturer’s instructions. The VECTOR NovaRED Peroxidase (HRP) Substrate Kit was used for visualization (Vector Laboratories, USA, Catalogue Number SK-4800). Nuclei of endothelial and lymphocytic cells in the slides served as positive internal control for BAP1 protein expression. The staining results were scored independently by three pathologists (HK, DC, WB). The percentage of positive tumor cell nuclei was scored only in areas with positive internal controls.

### Mutation analysis

Sanger sequencing was used for analysis of mutations in hotspot areas of *SF3B1* including codon 625 and codon 700 as well as for mutations in exon 1 and 2 and flanking intronic regions of *EIF1AX*. Primers are listed in Table 1. Normal tissue to exclude germline mutations was not available.

**Table 1:** Primers used for mutational analysis

Gene	Exon	Forward (Fw) Reverse (Rv)	Primer sequence 5′- 3′
*SF3B1*	14	Fw	TGATTATGGAAAGAAATGGTTGAAG
		Rv	AGGGCAATAAAGAAGGAATGC
*SF3B1*	15	Fw	TGCAGTTTGGCTGAATAGTTG
		Rv	GGCCAAAGCACTGATGGT
*EIF1AX*	1	Fw	CCCCTCGGAGCAGCAG
		Rv	CCTGGGTGACCTGCAATCTAC
*EIF1AX*	2	Fw	GCCTTAATTTCATTTTATTTCATACTGTTT
		Rv	AGGATGTTATTTTAAAAAGCGTAATTT

All primers contained a M13 forward or M13 reverse consensus sequence for sequencing. PCR amplification was performed in a total volume of 25 μl, containing 20 ng DNA, PCR Reaction Buffer with 20 mM MgCl2 (Roche), 200 μM of each deoxynucleotide triphosphate, 10 pmol of each primer and 2 units of FastStart Taq DNA polymerase (Roche). DNA amplification was performed in a Biometra T-Professional thermocycler (Westburg). The PCR was started with 5 min. at 95 °C and followed with 40 cycles of denaturation 30 s at 95 °C, annealing at 60 °C for 45 s and extension at 72 °C for 30 s, followed by a final extension at 72 °C for 10 min. and cooling down for 5 min. at 22 °C. All PCR products were purified with ExoSAP-IT (Affymetrix). Two μl of the PCR product was used for the sequence reaction on an ABI PRISM 3500XL DNA analyzer (Applied Biosystems). Both strands were sequenced using the M13 primers.

Information on *BAP1* mutation status was available for three patients (#20, #23, #24) (obtained by Sanger sequencing as previously described) while the chromosome 3 status was in part obtained from a previous MLPA study [22, 25]. For all cases, the mutation status of *GNAQ* and *GNA11* (codons 209 and 183), *BRAF* (codon 600) and *NRAS* (codons 12, 13 and 61) was available, part of this information has been published previously [2, 22, 23].

## Results

### Patients and histology

Main patient characteristics are summarized in Table 2. The study group consisted of thirteen melanocytomas, seven intermediate-grade melanocytic tumors and four primary leptomeningeal melanomas. A subset of the tumors was strongly pigmented.

**Table 2:** Patient characteristics and results of immunohistochemistry and mutation analyses

Patient	Sex	Age	Diagnosis	Location	BAP1 immunostaining	*Chrom 3*	*SF3B1* exon 14 (codon 625)	*SF3B1* exon 15 (codon 700)	*EIF1AX* exon 1	*EIF1AX* exon 2	*Follow*-*up*
1	F	27	MC	Right CPA	+ (80 %)	disomy	wt	wt	Intron c.1–4C > T	wt	local recurrence
2^a^	M	41	MC	C0-C3	+	disomy	wt	wt	c.9G > C (p.(Lys3Asn))	wt	local recurrence and LM seeding 3 years after diagnosis
3^a^	M	47	MC	Extramedullary, intradural	+	disomy	wt	wt	wt	na	local recurrence and LM seeding shortly after initial presentation
4^a^	M	27	MC	Tentorium cerebelli	+ (80 %)	na	c.1900G > A (p.(Val634Ile))	wt	wt	na	LM seeding shortly after initial presentation
5	M	38	MC	C5–6	+	na	wt	na	na	na	tumor spread in neck and vertebra; deceased
6^a^	M	41	MC	Th6	na	na	wt	wt	na	na	local recurrence; no distant metastasis
7^a^	V	57	MC	Th11	+	disomy	wt	wt	wt	wt	unknown
8^b^	F	62	MC	C0-C1	+	na	wt	wt	wt	wt	unknown
9	F	23	MC	Fossa posterior	na	na	wt	wt	wt	wt	local recurrence after 2 years
10^a^	M	55	MC	C3–6	na	na	wt	wt	wt	na	local recurrence 8 years after diagnosis
11^a^	M	na	MC	Spinal region	na	na	wt	wt	wt	wt	unknown
12	M	69	MC	Conus medullaris	+	na	wt	wt	wt	wt	alive
13	M	37	MC	Left CPA	+	na	wt	wt	wt	wt	unknown
14^b^	F	68	IMT	Tentorium cerebelli	+	disomy	wt	wt	c.11A > G (p.(Asn4Ser))	wt	deceased (not disease related)
15^a^	V	44	IMT	Cauda	+	disomy	wt	wt	wt	wt	stable (no recurrence)
16^a^	M	41	IMT	Intramedullary (NOS)	+	disomy	wt	wt	wt	c.25G > C (p.(Gly9Arg))	local recurrence and LM seeding
17^b^	V	59	IMT	Vermis cerebelli	+	na	wt	wt	wt	wt	unknown
18^b^	F	30	IMT	Th10–11	+	disomy	c.1873C > T (p.(Arg625Cys))	wt	wt	wt	leptomeningeal seeding; deceased (disease related)
19^a^	F	53	IMT	Th9	+	na	wt	wt	wt	c.28A > G (p.(Lys10Glu))	liver metastasis shortly after diagnosis
20^a,e^	F	48	IMT	Cervical spinal region	+	disomy	wt	wt	wt	wt	local recurrence after 3 years; distant metastasis to liver and pancreas 1 year later; deceased 5 years after initial presentation
21^c^	F	31	MM	Frontal left	+	disomy	c.1874G > A (p.(Arg625His)) ^d^	wt	wt		neurocutaneous melanocytosis patient; the *SF3B1* mutation was only present in the CNS melanoma and not in the congenital melanocytic nevus of the skin
22^a^	M	62	MM	Th7–9	+	na	na	na	c.9G > C (p.(Lys3Asn))	na	unknown
23^a,e^	F	59	MM	S2	+	Monosomy 3	wt	wt	wt	wt	distant metastases after 2 years (bone, lungs); liver metastasis unknown
24^a,e^	M	55	MM	L1-L2	+	na	wt	wt	wt	wt	leptomeningeal seeding 1 year after initial presentation; no distant metastases; deceased

Information on *GNAQ*/*GNA11* mutation status and chromosome 3 status of cases 1–7, 10, 13–18, and 22–24 has been published previously [2, 22, 23]

*F* female, *M* male, *MC* melanocytoma, *IMT* intermediate-grade melanocytic tumor, *MM* melanoma, *LM* leptomeningeal, na not available (BAP1 immunohistochemistry of cases #9–11) or not assessable, *CPA* cerebello-pontine angle

+ positive nuclear staining in 90 % or more of nuclei

^a^GNAQ^Q209^ or

^b^GNA11^Q209^ or

^c^NRAS^Q61^ mutation present

^d^the *SF3B1* mutation was not present in the congenital melanocytic nevus of this neurocutaneous melanocytosis patient

^e^cases for which all 17 exons of the *BAP1* gene were tested with Sanger sequencing and no mutations were detected

All patients were adults, the age at operation ranging from 27–69 years. None of the patients had clinical evidence of primary melanoma outside the CNS. One patient was known with a large congenital melanocytic nevus in the buttocks in the context of neurocutaneous melanocytosis (patient #21). Two of the patients in this series developed liver metastases, both after an initial histological diagnosis of intermediate-grade melanocytic tumor (patients #19 and #20). One melanoma patient developed distant metastases to bones and lungs (patient #23).

### BAP1 immunohistochemistry

**Fig. 1:**
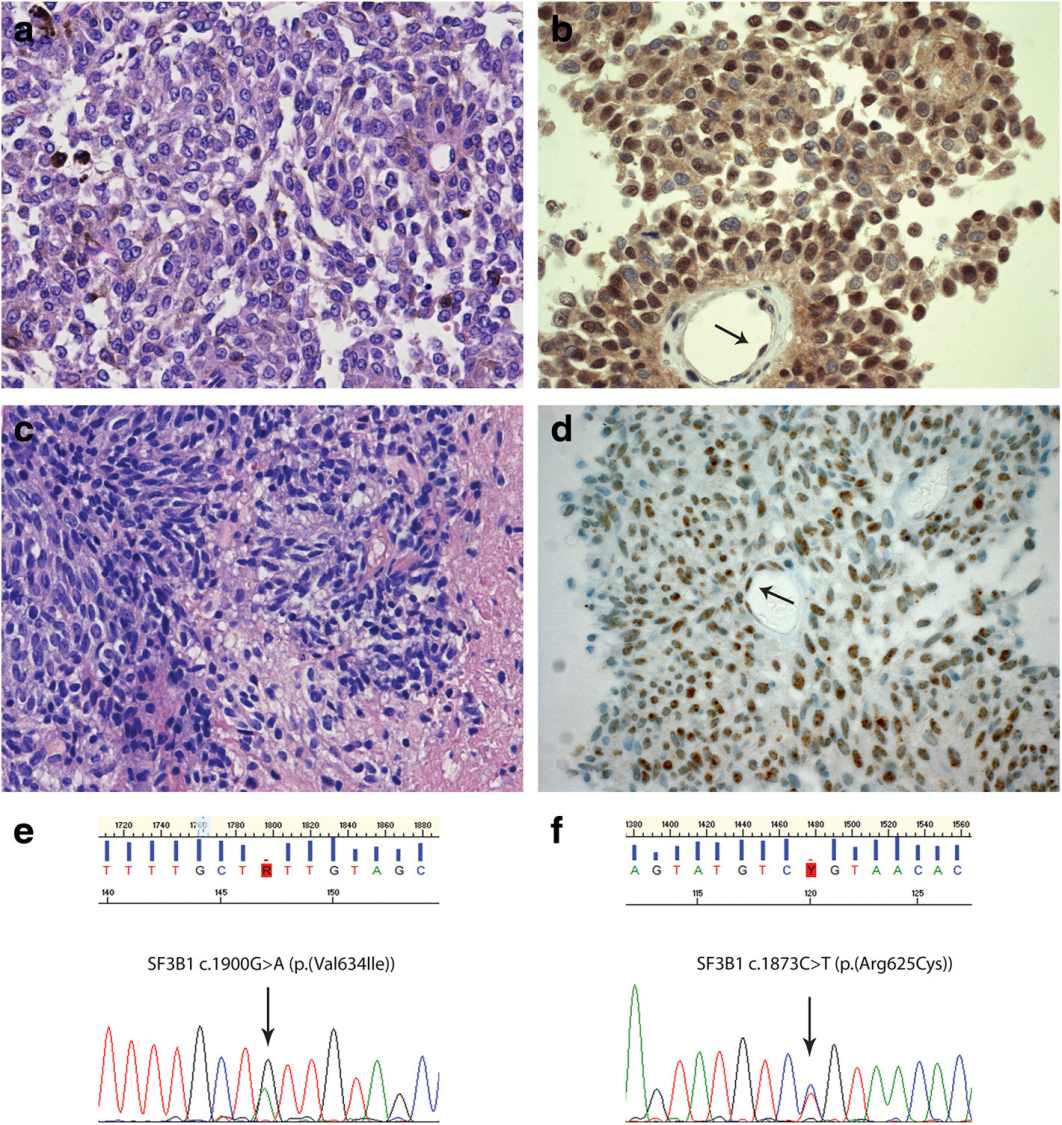
Examples of *SF3B1* mutation in a primary leptomeningeal melanocytoma and intermediate-grade melanocytic tumor. **a**. Hematoxylin-and-Eosin (H&E) staining of the melanocytoma of patient #4 showing round to oval, slightly pleomorphic, vesicular nuclei, often with a prominent nucleolus, and with dispersed melanin pigment in the cytoplasm of the tumor cells. **b**. BAP1 immunostaining of this melanocytoma revealed heterogeneous staining of the tumor cell nuclei, the majority of these nuclei being positive. The arrows indicate nuclear staining in endothelial cells. Note that the tumor cells also show variable cytoplasmic staining. **c**. H&E staining of the intermediate-grade melanocytic tumor of patient #18 showing a proliferation of spindle cells with invasion in glial tissue (at the right). **d**. BAP1 immunostaining of the tumor depicted in (**c**) reveals positive nuclei in more than 90 % of tumor cells. The arrows indicate nuclear staining in endothelial cells. **e**. Forward sequence tracing surrounding codon 634 of *SF3B1* showing the c.1900G > A (p.(Val634Ile)) mutation detected in the tumor depicted in (**a**) and (**b**). **f**. Forward sequence tracing surrounding codon 625 of *SF3B1* showing the c.1873C > T (p.(Arg625Cys)) mutation present in the tumor depicted in (**c**) and (**d**)

**Fig. 2:**
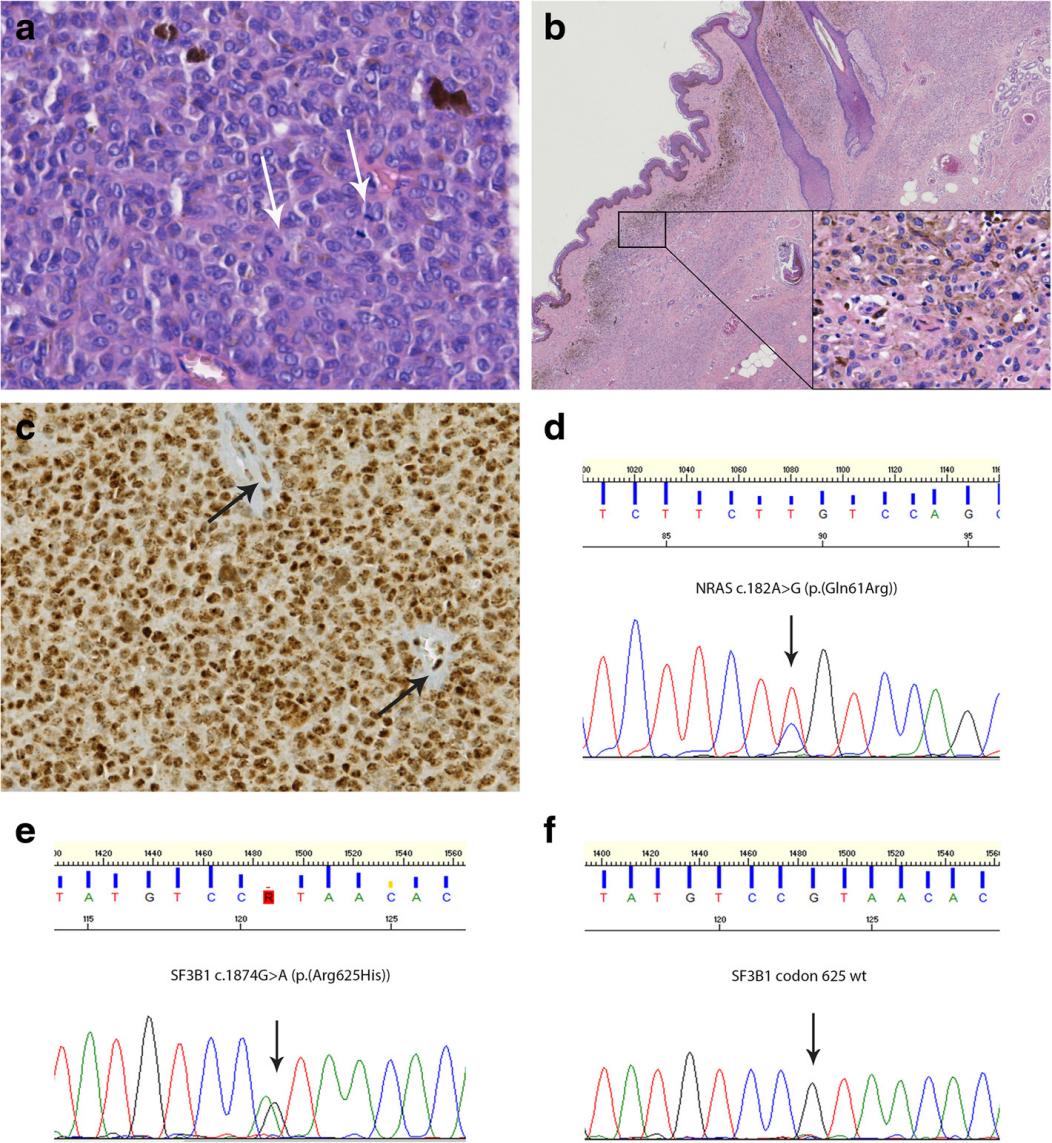
*SF3B1* mutation in primary leptomeningeal melanoma in a patient with neurocutaneous melanocytosis. **a**. H&E staining of the primary LMN diagnosed as melanoma; the arrows indicate mitotic figures (patient #21). **b**. H&E staining of the congenital melanocytic nevus of the buttocks; in the right lower corner higher magnification of the superficial part of the nevus with a proliferation of bland nevoid cells. **c**. BAP1 immunostaining of the melanoma depicted in (**a**) with nuclear staining in more than 90 % of tumor cells. **d**. Sequence tracing surrounding codon 61 of *NRAS* showing a c.182A > G (p.(Gln61Arg)) mutation present in both the CNS melanoma as well as in the congenital melanocytic nevus (reverse sequence/antisense strand). **e**. Forward sequence tracing surrounding codon 625 of *SF3B1* showing a c.1874G > A (p.(Arg625His)) mutation in the CNS melanoma. **f**. The *SF3B1* mutation depicted in (**e**) is absent in the congenital melanocytic nevus of this patient

**Fig. 3:**
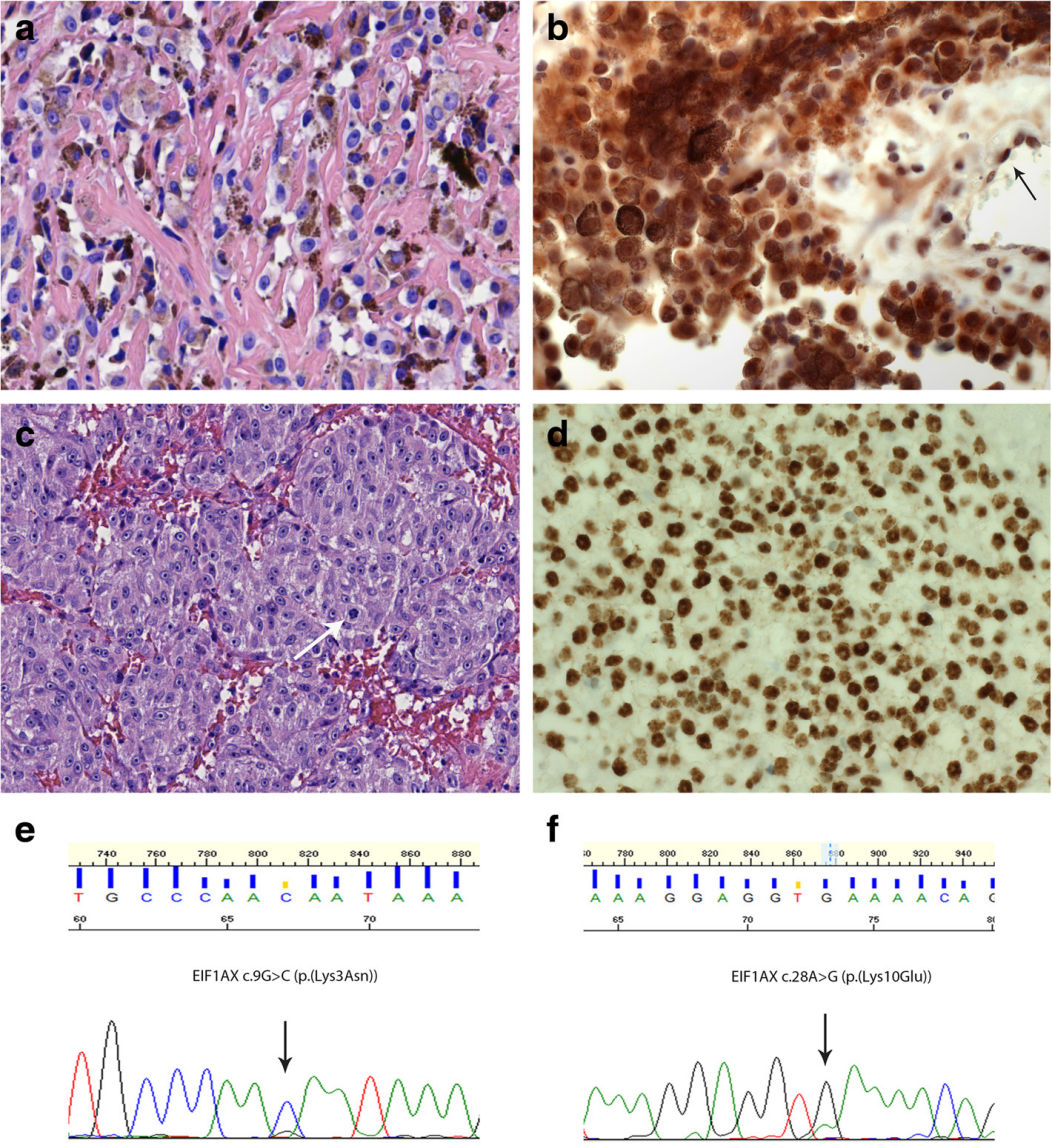
Examples of *EIF1AX* mutation in a primary leptomeningeal melanocytoma and intermediate-grade melanocytic tumor. **a**. H&E staining of the melanocytoma of patient #2, consisting of epithelioid cells with moderate to strong melanin pigmentation. **b**. BAP1 immunostaining of the melanocytoma depicted in A revealing positive staining of nuclei in more than 90 % of tumor cells. The arrows indicate nuclear staining in endothelial cells. Note that cytoplasmic staining is also present. **c**. H&E staining of the intermediate-grade melanocytic tumor of patient #19 showing a nested proliferation of epithelioid cells; the arrow indicates a mitotic figure. **d**. BAP1 immunostaining of the intermediate-grade melanocytic tumor depicted in C revealing positive staining of nuclei in more than 90 % of tumor cells. **e**. Forward sequence tracing surrounding codon 3 of *EIF1AX* showing a c.9G > C (p.(Lys3Asn)) mutation in the melanocytoma depicted in (**a**) and (**b**). **f**. Forward sequence tracing surrounding codon 10 of *EIF1AX* showing a c.28A > G (p.(Lys10Glu)) mutation in the intermediate-grade melanocytic tumor depicted in (**c**) and (**d**)

BAP1 immunohistochemistry was available in 20 out of 24 cases. For three cases there was no FFPE material left for immunohistochemistry (patients #9–11), while in one patient the tumor showed intense pigmentation, preventing reliable interpretation of staining results (patient #6). Only nuclear staining was considered as positive, although cytoplasmic staining was found in a substantial subset of tumors as well. In two melanocytomas (patients #1 and #4), a mosaic pattern with alternating positive and negative (areas of) nuclear staining was present, the vast majority (about 80 %) of these nuclei being positive (Fig. 1a, b). All other cases showed moderate to strong nuclear staining in 90 % or more of nuclei. Complete absence of nuclear BAP1 staining as is typically seen in *BAP1*-mutated tumors was not observed (Figs. 1, 2 and 3).

### Mutation analysis

We detected a total of three mutations in *SF3B1* (13 %), including two hotspot mutations at codon 625 (R625C and R625H) and one mutation affecting codon 634 (V634I) (Table 2). Mutations in codon 700 of *SF3B1* were not detected. In the cosmic database the V634I mutation in *SF3B1* (c.1900G > A (p.(Val634Ile))) has not been reported in cancer before (http://cancer.sanger.ac.uk/cosmic). This mutation was present in a *GNAQ*-mutated melanocytoma of a 27-year-old male patient who developed leptomeningeal seeding shortly after initial presentation (patient #4) (Fig. 1a, b, e).

One case with mutation in codon 625 of *SF3B1* (c.1873C > T (p.(Arg625Cys))) concerned an intermediate-grade melanocytic tumor showing aggressive behavior with leptomeningeal seeding (patient #18). A concomitant *GNA11* mutation was present in this tumor while immunohistochemistry showed intact nuclear BAP1 expression (Fig. 1c, d, f).

The other mutation in codon 625 of *SF3B1* (c.1874G > A (p.(Arg625His))) was present in a CNS melanoma of a 31-year-old woman (patient #21). Since birth this patient was known with a large congenital melanocytic nevus on the buttocks for which multiple excisions had been performed, confirming melanocytic nevus without malignant transformation. At the age of 29, she developed an intracranial tumor in the left frontal region with diffuse leptomeningeal extension. Biopsy showed melanoma in a background of diffuse melanocytosis, consistent with the diagnosis of primary melanoma of the CNS occurring in the context of neurocutaneous melanocytosis. This was supported by the presence of an *NRAS* mutation in both the CNS melanoma and in a more recent biopsy of the melanocytic nevus (c.182A > G (p.(Gln61Arg))). In contrast, the *SF3B1* mutation (c.1874G > A (p.(Arg625His))) was present in the CNS melanoma but not in the melanocytic nevus. Immunohistochemically, intact BAP1 protein expression was found in the tumor cell nuclei of this melanoma (Fig. 2).

A point mutation in *EIF1AX* was detected in five of the primary LMNs in this series (21 %), each leading to an amino acid substitution (at codons 3, 4, 9 or 10). These mutations occurred in tumors diagnosed as melanocytomas, intermediate-grade tumors, and melanomas (Fig. 3). In addition, one point mutation was detected in the Kozak consensus sequence of exon 1 (c.1–4C > T) which may have an influence on the start of translation (patient #1). *EIF1AX* mutations were mutually exclusive with *SF3B1* mutations and generally co-occurred with *GNAQ* or *GNA11* mutations (Table 2).

## Discussion

In this study we investigated whether primary LMNs share genetic alterations with UMs in addition to *GNAQ* and *GNA11* mutations. Recently, a role for *BAP1* in primary melanoma of the CNS was suggested based on the identification of a *BAP1* germline mutation in a patient with primary CNS melanoma with monosomy 3 and a family history of UM and meningioma [16]. In our study, we chose for BAP1 immunohistochemistry as a surrogate marker for the identification of an underlying inactivating *BAP1* mutation as it offers an economical and faster alternative to sequence analysis of all 17 exons of *BAP1* [12, 26, 27]. Typically, complete loss of nuclear BAP1 expression is found in *BAP1*-mutated UMs [12, 26–28]. None of the samples in our series showed such complete loss of nuclear BAP1 staining, suggesting absence of underlying *BAP1* mutations. In three cases (including the single patient with monosomy 3 in the LMN and the patient with liver metastases but disomy 3 in the LMN), *BAP1* mutation status was available through Sanger sequencing analyses and confirmed absence of mutations. However, our study has some limitations. In some cases, *BAP1* mutations may still have been missed as BAP1 immunohistochemistry was reported to have a sensitivity of ~ 88 % [12]. Also, very rarely, heterogeneous (‘mosaic’) BAP1 nuclear immunostaining has been described in UM cases with a *BAP1* mutation showing loss of nuclear staining in only 20 % of nuclei [12]. In our series, two cases showed nuclear BAP1 staining in about 80 % of tumor cells and an underlying *BAP1* mutation can thus not completely be ruled out. Furthermore, as *BAP1* is mainly implicated in metastatic UM with monosomy 3, there might be a selection bias in our patient group as it mainly concerns (relatively) low-grade tumors with disomy for chromosome 3. A larger number of primary LMNs with monosomy 3 should thus be investigated to further explore the role of *BAP1* in these neoplasms. This is also important for therapeutic reasons as epigenetic modulators such as histone deacetylase (HDAC) inhibitors were shown to reverse the biochemical effects of *BAP1* mutations in UM cells by inducing growth arrest and differentiation. Clinical trials are now evaluating HDAC inhibitors as a therapeutic option in UM patients [13, 29].

Recurrent mutations in the *SF3B1* gene have been detected in several types of cancer such as UMs, breast and pancreatic carcinoma, and hematological diseases like CLL and MDS [18, 30–33]. These mutations affect hotspot codons, the hotspot being associated with cancer type. For example, codon 700 mutations are frequently present in CLL and MDS, while in UMs codon 625 is much more frequently involved [18, 34]. Especially in low-grade UMs with disomy for chromosome 3, heterozygous mutations in *SF3B1* are present in 10 to 30 % of UM [6, 17–19]. The fact that in UMs these *SF3B1* mutations are almost mutually exclusive with *BAP1* mutations suggests different pathways in the oncogenesis and/or malignant progression of these neoplasms. *SF3B1* encodes subunit 1 of splicing factor 3b, which is a component of the spliceosome that participates in splicing of pre-mRNA. It was shown that *SF3B1* mutations are associated with differential alternative splicing of several protein encoding genes in UMs [19]. Moreover, *SF3B1* mutant cell lines were found to be sensitive to the SF3b complex inhibitor spliceostatin A, suggesting a new therapeutic target in tumors carrying this mutation [32]. Up to now, it was unknown whether *SF3B1* mutations also occur in primary LMNs. In our series, in three out of 24 cases (13 %) an *SF3B1* mutation affecting codon 625 or 634 was detected, this is at the lower end of the range of the frequency of *SF3B1* mutations reported in UMs (10–30 %) and in contrast to the very low frequency reported for CMs (~1 %) [6, 17–20]. As far as could be assessed, all three LMNs showing an *SF3B1* mutation were tumors with disomy for chromosome 3. Furthermore, like in UMs, in two cases the *SF3B1* mutation co-occurred with a *GNAQ* or *GNA11* mutation, while in a third case (with neurocutaneous melanocytosis) an *NRAS* mutation was present. The *GNAQ*, *GNA11* and *NRAS* genes are now thought to play a role in the initiation of tumorigenesis, while mutation in *SF3B1* (or *BAP1*) would then occur in a later phase of the oncogenic process [18]. The co-occurrence of an *NRAS* and an *SF3B1* mutation in the CNS melanoma of the neurocutaneous melanocytosis patient in our study is interesting in this respect as the *SF3B1* mutation was absent in the congenital melanocytic nevus of this patient. Patients with neurocutaneous melanocytosis have a large and/or multiple congenital melanocytic nevi of the skin in association with a primary LMN [1]. Instead of *GNAQ* or *GNA11* mutations, these patients frequently demonstrate identical *NRAS* mutations in both the congenital melanocytic nevus and in the LMN [35, 36]. This is thought to be the result of an *NRAS*-mutated clone of melanocyte precursors migrating to skin and CNS early in embryogenesis [37]. The observation that the *SF3B1* mutation was not present in the melanocytic nevus of this patient suggests that it indeed plays a role later on in tumorigenesis. In addition, all three mutations in *SF3B1* in our study were found in tumors which clinically showed aggressive behavior. However, the number of patients and the follow-up in our study are too limited to allow for firm conclusions about a prognostic role of *SF3B1* in this setting. Of note, in one of the three patients the *SF3B1* mutation (c.1900G > A (p.(Val634Ile))) was present in a neoplasm diagnosed as melanocytoma, suggesting that *SF3B1* mutations are not necessarily associated with worrisome histology.

Several recent studies have reported mutations in *EIF1AX* in different cancer types, including melanoma and thyroid and ovarian cancer [17, 38, 39]. In UMs, heterozygous mutations in exon 1 and 2 of *EIF1AX* have reported to occur especially in tumors with disomy for chromosome 3 (up to 48 % of these tumors) [17]. Mutations may occur in different loci of these exons and lead to amino acid substitutions or short deletions. Rarely splice site mutations have been reported [6, 10, 17]. *EIF1AX* encodes the eukaryotic translation initiation factor 1A (eIF1A), which is involved in initiation phase of translation of eukaryotic cells by stabilizing the formation of the functional ribosome around the AUG start codon. The exact role of *EIF1AX* mutations in tumorigenesis is currently not well understood but it has been suggested that mutations in *EIF1AX* could diminish the rate of bulk translation [17, 40]. In our series of primary LMNs we found a relatively high frequency of *EIF1AX* missense mutations (21 %) which is in the range reported for UM (19–48 %) [6, 17]. In contrast, *EIF1AX* mutations are very rare in CM (5/231, ~2 %) [20]. As in UMs, as far as could be assessed in our series, these mutations occurred in primary LMNs with disomy for chromosome 3 and were mutually exclusive with *SF3B1* mutations, but co-occurred with *GNAQ* or *GNA11* mutations. In addition (and like *SF3B1* mutations), the *EIF1AX* mutations in our cases occurred both in melanocytomas as well as melanomas, suggesting that they are not necessarily associated with worrisome histology, but the prognostic implications of these mutations remain to be elucidated. Finally, the *EIF1AX* and *SF3B1* mutations in LMNs occurred in hotspot regions of these genes, and in UMs such mutations were shown to be somatic in origin [17, 18]. However, as non-neoplastic tissue of the patients with LMNs was not available for further testing, strictly speaking we cannot rule out the possibility that in some of these cases it concerned a germline mutation.

## Conclusions

We report for the first time that a substantial subset of LMNs carries a mutation in *SF3B1* or *EIF1AX*. Like in UMs, *SF3B1* or *EIF1AX* mutations are mutually exclusive and generally co-occur with either a *GNAQ* or *GNA11* mutation (*SF3B1* occasionally with *NRAS* mutation), suggesting that *SF3B1* and *EIF1AX* mutations occur later on in the tumorigenesis. Our findings may offer novel therapeutic strategies for especially patients with *SF3B1*-mutated LMNs. The role of *BAP1* in the pathogenesis of primary LMNs is less clear, immunohistochemistry suggests that *BAP1* mutation is infrequent in tumors with disomy for chromosome 3. However, more detailed analysis is needed for further elucidation of the role of *BAP1* in the oncogenesis of primary LMNs. Demonstration of *BAP1* mutations in primary LMNs would potentially have therapeutic relevance as well, as epigenetic modulators are now being evaluated in patients with *BAP1*-mutant UMs. All in all, this study thus underscores the genetic resemblance of primary LMNs and UMs and provides some additional clues for the diagnosis of and therapeutic interference with these tumors.

## Abbreviations

CNS: Central nervous system
CM: Cutaneous melanoma
FFPE: Formalin fixed and paraffin embedded
HDAC: Histone deacetylase inhibitors
LM: Leptomeningeal
LMN: Leptomeningeal melanocytic neoplasms
UM: Uveal melanoma
